# Cutaneous Metastasis Following a Six-Year History of Bladder Urothelial Carcinoma in Situ: A Case Report

**DOI:** 10.7759/cureus.61193

**Published:** 2024-05-27

**Authors:** Jamila Mammadova, Rasika Patil, Timothy Herman, Vanessa I Rodriguez

**Affiliations:** 1 Department of Internal Medicine, University of South Florida Morsani College of Medicine, Tampa, USA

**Keywords:** case report, carcinoma in situ, bladder cancer, transitional cell carcinoma, urothelial carcinoma, cutaneous metastasis

## Abstract

Bladder cancer with cutaneous metastasis is a rare manifestation of the advanced stage of the disease. It can result from direct invasion, lymphatic or hematogenous spread, or iatrogenic implantation. We present a case of a 67-year-old patient initially diagnosed with urothelial carcinoma (UC) in situ of the bladder, who underwent transurethral resection of bladder tumor, along with induction and maintenance Bacillus Calmette-Guerin immunotherapy. Six years post-diagnosis, the patient developed multiple ulcerating fungating lesions in the right lower extremity, confirmed as metastases from UC. The patient additionally developed right foot gangrene with subsequent infection, which progressed into sepsis and caused the patient’s demise.

## Introduction

Bladder cancer ranks as the sixth most prevalent malignancy in the United States with a five-year survival rate of merely 5% when diagnosed in its metastatic stage [1]. Roughly 90% of bladder cancers originate from urothelial cells, which line the bladder and get in contact with various potential toxins and mutagenic agents. The disease commonly metastasizes to lymph nodes, bones, lungs, liver, and peritoneum [2]. It can also spread to the skin, an exceptionally rare occurrence called cutaneous metastasis, with an incidence rate of 0.84% [3]. Herein, we present a case involving a 67-year-old male who developed cutaneous metastasis of bladder cancer six years post-treatment for bladder carcinoma in situ (CIS). This is the first reported case of cutaneous metastasis from CIS of the urothelial carcinoma (UC) of the bladder.

## Case presentation

We present a case of a 67-year-old male, a former 15-pack-year smoker who ceased smoking 27 years before the presentation. He initially presented with intermittent gross hematuria, and a computed tomography (CT) scan revealed a bladder lesion. Cystoscopy with cytology and bladder biopsy revealed atypical cells and low-grade papillary UC at the trigone and high-grade UC at the bladder dome. The patient underwent transurethral resection of bladder tumor (TURBT) within one month after the diagnosis. The post-surgical pathology was consistent with CIS. The patient completed a Bacillus Calmette-Guerin (BCG) bladder induction course of six weeks. After the induction, the patient experienced recurrent hematuria with atypical cells on cytology, though repeat cystoscopy with biopsy was negative for malignancy. Subsequently, the patient completed three courses of maintenance BCG therapy, after which a repeat cystoscopy with biopsy identified no CIS or invasive carcinoma. He was then lost to follow up with his maintenance and surveillance. 

**Figure 1:**
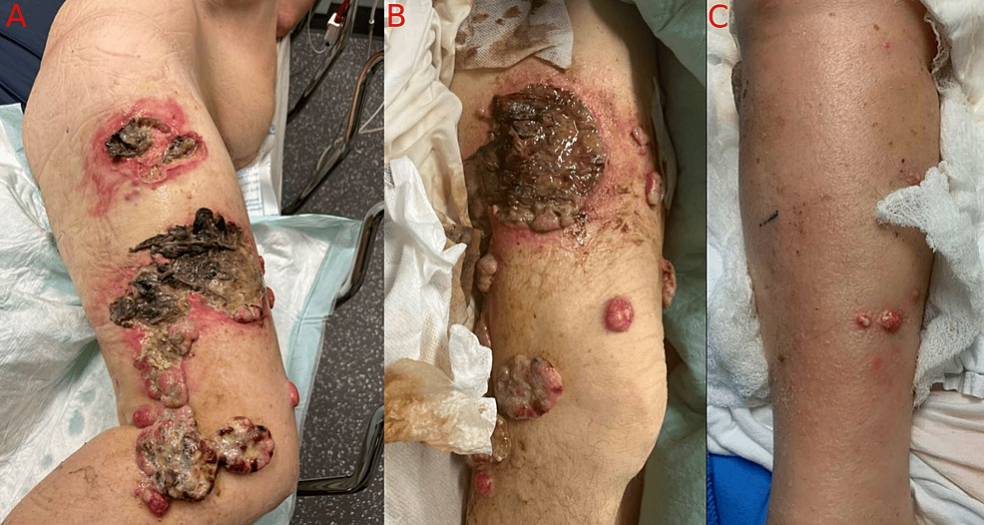
Nodular fungating cutaneous lesions of the right thigh (A, B) and the right shin (C)

Six years later, the patient presented with a five-month history of multiple lesions along his entire right lower extremity, which he initially attributed to a spider bite (Figure 1). These lesions progressed to become fungating, exuding clear serous fluid with minor bleeding, and causing worsening pain, the worst in the foot, which severely limited ambulation. Additionally, the patient reported an 80-pound weight loss over six months.

Biopsies of the lesions revealed poorly differentiated carcinoma with squamous differentiation and fibroconnective tissue. The neoplastic cells were positive for pankeratin, p63, CK5/6, p16, and GATA3, while CK7 was negative. The pathology results favored metastatic urothelial carcinoma with squamous differentiation. Metastatic squamous cell carcinoma of the anogenital region would have a similar histologic and immunohistochemical profile, which was excluded with colonoscopy.

**Figure 2:**
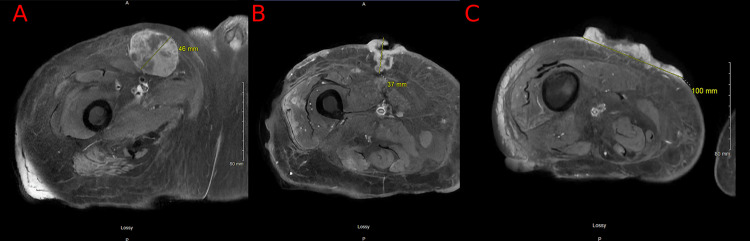
Magnetic resonance imaging of the right femur showing enlarged right inguinal lymph nodes (A) and deep skin and subcutaneous soft tissue lesions in the right thigh (B, C)

Radiologic workup, including CT scans of the neck, chest, and abdomen, showed bilateral cervical, mediastinal, inguinal, iliac, and periaortic adenopathy. Magnetic resonance imaging (MRI) of the right femur showed multiple subcutaneous soft tissue lesions in the right thigh along with a large ulcerated soft tissue area (Figure 2). A biopsy of lymph nodes showed metastatic carcinoma with squamous morphology. Histologic sections of the core biopsy showed infiltrating nests of pleomorphic tumor cells with squamous morphology consistent with recent surgical skin biopsy findings from the right thigh. A primary gastrointestinal tumor was ruled out with a colonoscopy. Evaluation by the oncology team concluded that the patient would not be a candidate for chemotherapy due to the poor Eastern Cooperative Oncology Group (ECOG) performance status of 3-4 and recommended considering immunotherapy in an outpatient setting. The patient received one dose of cemiplimab. For the management of the bleeding from the cutaneous lesions, the patient underwent two radiation treatments.

**Figure 3:**
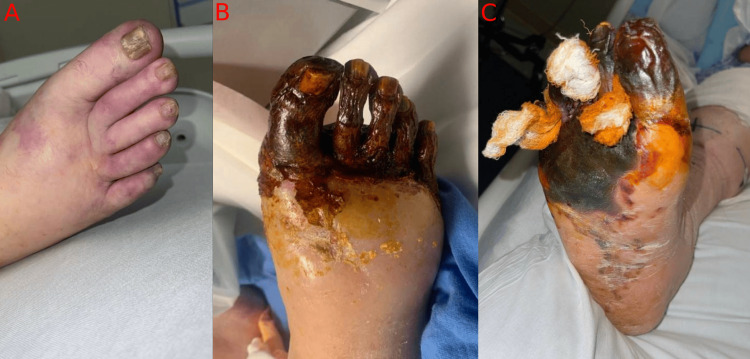
Ischemia-in-the-right-foot-(A)-that-progressed-to-wet-gangrene-(B,-C)

The patient also developed an acute-on-chronic thrombus of the common femoral artery, surgically removed, likely contributing to right foot gangrene (Figure 3).

The right foot gangrene was colonized with pseudomonas and was treated with IV antibiotics. Due to persistent infection, an amputation after immunotherapy was recommended. The patient developed superinfection of necrotic malignancy, leading to a decrease in mean arterial pressure (MAP), development of septic shock, and eventual demise despite aggressive management.

## Discussion

Cutaneous metastasis in UC of the bladder is a rare occurrence in advanced disease with a reported incidence of 0.84% [3]. These lesions may present concurrently with the primary cancer diagnosis or develop months to years after initial treatment. Previously, only a handful of cases of cutaneous metastasis in UC were documented in the literature, all associated with invasive malignancy at the time of primary diagnosis. However, in our case, the biopsy findings indicated CIS six years before the development of cutaneous metastases, marking it as the first reported instance of cutaneous metastasis from CIS in UC.

The pathophysiology of cutaneous metastases from UC can be secondary to direct invasion from the bladder, lymphatic or hematogenous spread, or iatrogenic implantation, with the latter being the most common cause [4]. A radiologic evaluation of our patient revealed widespread systemic lymphadenopathy, which was biopsied and consistent with metastatic urothelial malignancy, suggesting lymphatic spread. While most cases in the literature occurred due to iatrogenic seeding, an extended time lapse between UC treatment and the development of cutaneous metastasis suggests systemic spread, as primary cutaneous metastasis is a late manifestation of the systemic spread of the disease [5].

Diagnosis of cutaneous metastasis from UC can be challenging at the initial presentation as the gross appearance of the lesions can be consistent with skin malignancies, non-neoplastic skin conditions, or metastatic anogenital cancers. The gross morphology of cutaneous metastases is not distinct; it can be fibrotic, nodular, or inflammatory [6]. In our case, the presentation was consistent with nodular fungating lesions with ulceration and minor bleeding. A biopsy aids in further diagnosis, but clinical correlation and thorough diagnostic workup are essential to rule out primary skin cancers or anogenital malignancies. In our case, anogenital cancer was excluded with colonoscopy. Clinical course, radiologic findings, and immunohistochemistry characteristics, including positive p63, GATA3, and cytokeratin, supported urothelial origin with squamous differentiation over squamous cell carcinoma of the skin [7]. Expression of antigens in UC cells differs between invasive and non-invasive malignancies. For poorly differentiated invasive UC, expression of CK7, CK20, and p63 is common, but is not always present. When CK7 and CK20 are absent, it is common to observe the expression of p63 [7]. Markers described in clinical use include CK5/6, CK14, CK20, GATA3, p53, Ki-67, and uroplakin II. However, novel biomarkers were also reported in the literature [8]. GATA3 can be used to distinguish pure UCs and UCs with squamous differentiation from primary squamous cell carcinomas, although these tumors do not always display reactivity to individual markers corresponding to morphologic differentiation [9].

Cutaneous metastasis from UC is exceedingly rare, with only 25 cases reported between 2000 and 2023 [10-12]. Most cases developed cutaneous metastasis within the first year after UC treatment, with only two instances exceeding a three-year interval between primary diagnosis and cutaneous metastasis. Our case presented six years post-primary treatment, comprising TURBT, induction, and maintenance immunotherapies. The literature review showed that, in most cases (23/25), UC was invasive at primary diagnosis. The remaining two reports did not indicate the tumor invasiveness [13,14]. Our case highlights the need to consider cancer etiology in cutaneous lesions even in CIS malignancy years post-treatment. Unfortunately, our patient delayed seeking evaluation of skin lesions for five months, attributing them to spider bites, which likely contributed to the advanced disease state upon diagnosis.

The clinical implications of UC with cutaneous metastasis are grave, with median survival ranging from 13 to 14 months with chemotherapy or less than 12 months overall, given their poor response to treatments [10,15]. However, in cases with a solitary lesion, local excision, followed by chemotherapy, may offer potential curative outcomes despite advanced stages at diagnosis [16]. In our case, multiple widespread cutaneous lesions rendered them unresectable. Right food gangrene, likely secondary to arterial thrombus, further complicated our case. Palliative treatments with chemotherapy or immunotherapy can be considered depending on the patient's performance status, though, sadly, our patient passed away before treatment initiation.

## Conclusions

Cutaneous metastasis of UC is rare, with no previously reported cases in patients initially diagnosed with CIS of UC. Timely recognition and thorough evaluation of skin lesions are essential for prompt diagnosis and potential intervention. The prolonged time interval between CIS treatment and cutaneous metastasis in our case emphasizes the importance of vigilant surveillance even after years of disease remission. The unfavorable prognosis of this condition may be attributed to its advanced stage at diagnosis, coupled with its aggressive behavior and the limited treatment modalities available.
